# Gender-Specific Aspects of Health Literacy: Perceptions of Interactions with Migrants among Health Care Providers in Germany

**DOI:** 10.3390/ijerph17072189

**Published:** 2020-03-25

**Authors:** Digo Chakraverty, Annika Baumeister, Angela Aldin, Tina Jakob, Ümran Sema Seven, Christiane Woopen, Nicole Skoetz, Elke Kalbe

**Affiliations:** 1 Medical Psychology | Neuropsychology and Gender Studies & Center for Neuropsychological Diagnostics and Intervention (CeNDI), Faculty of Medicine and University Hospital Cologne, University of Cologne, 50937 Cologne, Germany; uemran.seven@uk-koeln.de (Ü.S.S.); elke.kalbe@uk-koeln.de (E.K.); 2 Research Unit Ethics, Faculty of Medicine and University Hospital of Cologne, and Cologne Center for Ethics, Rights, Economics, and Social Sciences of Health (CERES), University of Cologne, 50931 Cologne, Germany; annika.baumeister@uk-koeln.de (A.B.); Christiane.woopen@uk-koeln.de (C.W.); 3 Evidence-Based Internal Medicine, Department I of Internal Medicine and Center for Integrated Oncology Aachen Bonn Cologne Duesseldorf, Faculty of Medicine and University Hospital Cologne, University of Cologne, 50935 Cologne, Germany; angela.aldin@uk-koeln.de (A.A.); tina.jakob@uk-koeln.de (T.J.); Nicole.skoetz@uk-koeln.de (N.S.)

**Keywords:** gender, migration, health literacy, qualitative content analysis, health care professionals

## Abstract

Health literacy can be described as a complex process shaped by individual resources and preferences and by the nature and quality of health-related information people encounter. The main objective of this study was to explore the views of health care professionals on how gender as a personal determinant of health literacy affected their interactions with migrant patients. The interrelated challenges, needs and applied solutions were analyzed from a health literacy perspective. Five focus group discussions with health care professionals working with migrants (*n* = 31) were conducted in Cologne, Germany, audio recorded, transcribed and analyzed by qualitative content analysis. Gender-specific aspects, such as the gender of health care providers as a factor, were portrayed above all in relation to patients from Turkey and Arab countries regarding access to and understanding of health-related information. These statements exclusively represent the possibly biased or assumptions-based perspectives of health care professionals on their migrant patients and were made against the background of a systemic lack of time and the challenge of overcoming language barriers. Especially in this context, reducing time pressure and improving communication in the treatment setting may be to the benefit of all actors within healthcare.

## 1. Introduction

Health care can broadly be defined as the entirety of measures and activities promoting the health of human beings on a community or individual level [1]. The opportunities for achieving optimal health vary between different groups of people, with structural and social determinants influencing access to health care services and interactions between patients and health care providers. In order to gain a deeper understanding of these interactions it is important to look at factors shaping health opportunities.

The terms sex and gender originally used to be synonyms, both applied to indicate whether a person was male or female [2]. After Simone de Beauvoir’s seminal work The Second Sex in 1949 [3] the debate about the social constructiveness of being a man or woman led to the term gender now widely used for gender role. In contrast, the word sex usually serves for the biological distinction between male and female persons [4]. As of today, gender and queer theory has evolved far beyond the man-woman dichotomy [5,6]. Still, it is important to look at differences between men and women as the social and societal roles associated with these genders are important factors regarding individual health. For example, men and women partly suffer from different diseases and deal with them in different ways, which demands gender-sensitive diagnostic and therapeutic techniques [7,8]. This is even more so because gender also influences the way patients are diagnosed and treated by medical personnel [9]. Gender roles, gendered power relations, religious and cultural understandings of sexuality, and gender-specific access to educational resources can vary between the world’s regions [10,11,12]. As the number of transnational migrants has risen to an estimated 258 million in 2017 [13], a growing number of persons with different understandings of gender encounter each other within the health care systems of the host countries.

In the recent history of Germany there have been several phases of intensive immigration, the most recent one concerning refugees mainly from Syria and Iraq in 2015 [14]. In the 1950s, massive numbers of workers from Italy, Greece and above all Turkey were recruited to work in the factories of the up-and-coming German industry [15]. Although it was planned that these workers would return to their home countries, many of them decided to stay in Germany with their families. As a statistical category, the term “persons with a migrant background” has subsequently become established as a term for people who themselves or at least one of their parents were born without German nationality. This accounts for around 25% of the German population [16].

From a gender perspective, the interaction between migrants and the representatives of health care systems (e.g., health care providers) can be a challenging task for both sides. The gender of patients and physicians has been shown to influence doctor-patient interaction [17,18,19], and cross-cultural interactions have been described as demanding by patients and health care professionals (HCPs) [20]. The exchange of health-related information is a central aspect of the treatment situation [21]. In this regard, the ability to handle health-related information is an important factor—an ability neatly tied to the concept of health literacy.

Health literacy, a term first coined in the 1970s [22], has since been defined in numerous ways [23]. In 2012, Sørensen et al., proposed an integrated conceptual model of health literacy, reconciling 17 definitions and 12 models of health literacy [24]. Drawing on this integrated model, health literacy is defined as “the knowledge, motivation and competencies of accessing, understanding, appraising and applying health-related information within the healthcare, disease prevention and health promotion setting, respectively” [24]. Importantly, the model describes health literacy as a social-relational concept with societal, environmental, situational and personal determinants influencing a person’s health literacy. Gender can be understood (and is described by Sørensen et al.,) as a personal determinant for health literacy [24] with numerous societal, environmental and situational connotations that go far beyond biological sex differences. Migration can also be integrated into the model in several ways: Having a migrant background as a personal determinant, the migration process as a situational factor, both also connecting to societal and environmental aspects that may differ between host countries.

In a recent representative study conducted in Germany, 54% of the German population indicated to have limited health literacy while with a migrant background it was 71% of persons. This is in line with international studies comparing migrant’s health literacy with that of the general population [25]. Considering overall health literacy, correlations have been found between health literacy scores and gender [26,27,28,29,30]. However, the strength and the direction of the effects found in these studies are highly inconsistent and do not allow for a derivation of conclusive statements. It is still unclear how and in which direction gender aspects affect health literacy, especially in persons from culturally diverse backgrounds.

Within the health care systems, encounters of persons with a migrant background and HCPs typically take place in a treatment setting, with an HCP representing and acting on behalf of the health care systems of the receiving countries. HCPs work at the focal point of health literacy, where health information is obtained, understood, appraised and applied. Many of them interact with men and women of numerous different origins. The exploration of experiences from their everyday work has the potential to help in gaining a more profound understanding of how gender may affect health literacy in cross-cultural encounters in health care. There is a growing body of research on health literacy in the context of migration [31], and gender aspects in providing health care for migrants are slowly receiving attention [32]. However, relating the influence of gender-specific aspects of interactions between HCPs and migrants to the concrete steps of processing health information is a new approach which might help to comprehend the role of gender in this context.

It is important to note that this study cannot provide “objective” data on migrants and their health literacy. It can only offer the HCPs’ subjective perspective on the health literacy of migrants derived from their interactions with them in the treatment setting.

This research is part of a an overarching project regarding Gender-Specific Health Literacy in Individuals with Migrant background (GLIM) which consists of systematic reviews [33,34,35] and a further qualitative analysis concerning organizational health literacy which is not within the scope of this study. The main objective of this study was to explore the views of health care professionals on how gender as a personal determinant of health literacy may affect their interactions with migrant patients. The interrelated challenges, needs, and applied solutions were analyzed from a health literacy perspective.

## 2. Materials and Methods

### 2.1. Focus Group Discussions (FGD)—Method and Ethical Clearance

For explorational research questions, the choice of qualitative methods is recommended [36]. An FGD is a qualitative method frequently used in health research and education as “a research technique that collects data through group interaction on a topic determined by the researcher” [37]. FGD can be used to obtain sufficient information within a short time to determine the participants’ perspectives on a topic [38]. It is a moderated discussion procedure in which small groups are stimulated to discuss a given topic by means of an information input. Current studies show that with the implementation of two to three FGDs, usually at least 80% of the topics to be explored can be covered [39]. In this study, five FGD had to be conducted until saturation was reached with regard to the categorized responses. The study was approved by the Ethics Committee of the Medical Faculty of the University of Cologne (n 17-406).

### 2.2. Guideline Development

A guideline for the FGD was developed including the starting question and a set of probing questions for deepening topics or steering the conversation to aspects not yet mentioned in the respective discussion [40]. For the purpose of pretesting, two FGD were conducted with researchers from the department of Medical Psychology and CERES (Cologne Center for Ethics, Rights, Economics, and Social Sciences of Health) of the University of Cologne. In these FGD, the guideline was tested for consistency and structure. After the pretests, the format including the guideline, moderation and setting (guideline, length of discussion, seating order) were discussed with the participating researchers.

### 2.3. Participant Recruitment

Participants were recruited via purposive and snowball sampling. At first, practices and institutions listed in the Health Guide for Migrants [41], provided by the city of Cologne, were contacted via email and telephone. The guide is an electronic document which includes a list of health care institutions (hospitals, pharmacies) and practices (medical doctors, physiotherapists, midwives etc.,) which offer multilingual services. Further participants were recruited through online search, professional contacts of the researchers, and by putting a call for participation on the intranet message boards of hospitals in Cologne and the surrounding area. We included participants with a degree or certificate in a health-related profession who had been working with patients with a migrant background on a regular basis for at least two years. HCPs signaling general interest in taking part in one of the FGD received further information about the study as well as a written consent form including a data protection agreement. The material was sent to those willing to participate by e-mail or in written form. To avoid uneasiness, participants sharing a hierarchical work relation or working in the same department did not take part in the same FGD. Additional participants were recruited until saturation was reached [42].

### 2.4. Implementation of the FGD

At the beginning of every FGD, the participants received information material for the study and a socio-demographic questionnaire containing questions about their gender, migrant background and occupation. They were given sufficient time to read the material before signing the informed consent. Three researchers were present throughout the discussions with one being the moderator while the other two researchers posed additional probing questions in case they felt the need to dive deeper into a topic. A research assistant wrote a protocol in order to simplify the assignment of statements to the participants when transcribing the audio recordings. Two audio recording devices were used to avoid data loss.

The researchers introduced themselves and shortly explained the study purposes. The stimulus was set by introducing the concept of health literacy verbally and visually (in a poster format), the project-specific definition of a migrant background and gender as a personal determinant of health literacy. Following this, conversation recommendations were announced by the moderator including the request not to interrupt other participants and to treat everything said in the FGD as confidential. The participants were encouraged to elaborate on their own experiences, may whether they were in line with those of the other participants or not. The audio recordings were started before the participants introduced themselves, shortly describing the context in which they usually interacted with persons with a migrant background on a professional basis. After the introductions, the moderator invited the participants to share their experiences as follows: *“Well, you all work in a health care context with women and men who have a migrant background. Please take three minutes time to remember concrete situations from your day-to-day work, for example a treatment situation with the persons themselves or with their relatives, that was very typical or maybe even special and which you still have vivid memories of - regardless of whether it was solved satisfactorily. You are also welcome to take notes on this.”* After three minutes, the discussion was opened by the moderator. Probing questions were set to examine the needs and applied solutions that arose from the situations described (e.g., *“How satisfied were you with the outcome of the situation?”* or *“What did you miss in this situation and what would you have needed to meet the challenge?”*). If situations were described that only affected one gender, the HCPs were asked to talk about similar situations with other genders involved (“*Does anyone else in this group have experiences regarding this kind of situation when treating male/female patients?*”). If gender-specific aspects were not mentioned during the discussions, additional probing questions were posed to gently encourage the participants to consider potentially relevant gender aspects (e.g., “*What role did your own gender play in this situation*?”).

Every FGD reached the maximum of 120 min. At the end of each FGD, participants were asked for their opinion regarding the discussion, its format and what they felt needed improving. They were offered to receive information about the results. Participants received a reimbursement of 25 €.

### 2.5. Data Analysis

The audio records were transcribed verbatim. A qualitative data analysis software MAXQDA [43] was used to analyze the transcripts in the German language. Quotes displayed throughout this manuscript were translated and back-translated by a researcher fluent in English.

Following the recommendations for qualitative content analysis according to Kuckartz [44], a combination of deductive application of categories and inductive development of categories was performed by the involvement of two researchers (D.C. and A.B.). First, three main categories were deductively derived from the research question including perceived Challenges, Needs and Applied Solutions related to the treatment and care of people with a migrant background. In a second step, according to the guiding framework [24], additional deductive categories were applied including the four steps of health information processing Access, Understand, Appraise and Apply health information and subordinated to each of the three main categories to ensure that all inductively evolving subcategories related to health literacy could be identified. Inductive subcategories that were considered to be directly or indirectly related to Gender as a personal determinant of health literacy were exclusively derived from the data. Other inductive subcategories that arose from the text were subordinated to the three main categories Challenges, Needs, and Applied Solutions whenever possible without a considerable loss of information.

### 2.6. Reliability and Validity

Two researchers (D.C. and A.B.) independently coded the first FGD transcript, each researcher building inductive categories and subcategories of the deductive categories. In a second step, the two category systems were integrated into one. The two coding researchers then independently coded each of the remaining four FGD transcripts based on the preliminary category system, again reconciling and reflecting on the deductively and inductively derived main- and subcategories after a phase of independent coding. All potential discrepancies were resolved by consulting the mediating researcher (A.A.), who was highly involved in the whole research process. This research followed The Consolidated Criteria for Reporting Qualitative Studies [45].

## 3. Results

Between January 2018 and May 2019 we conducted five FGD with *n* = 31 participants at CERES. An overview of the characteristics of the participants is presented in Table 1.

The classification of the statements in this scheme shows which processing steps of health literacy were primarily influenced by gender. This could occur in two ways: (a) as a direct influence, described by gender subcategories or (b) as an indirect influence, which is represented by general subcategories. An overview of the most important categories is shown in Table 2.

### 3.1. Narrative Elements Used by the HCP

#### 3.1.1. Specific Situations, Generalizations and Possible Biases

The statements of the HCP often related to specific situations that were meant to act as examples for challenges, needs and applied solutions when interacting with migrants. This must be seen with the caveat that the selection and description of these situations may give a biased picture of the interaction with migrants, as stereotypes about migrants are very common in the general population, including HCPs [46]. When a phenomenon was perceived to occur frequently, the HCP talked about it in a more general way. Generalizations, especially about minority groups, are particularly prone to be stereotypical. As stereotypes can be internalized by members of the stereotyped group as well [47], this reservation accounts for the statements of all HCPs including those who themselves were first or second generation migrants. Therefore, the statements should be regarded as subjective and selective narratives.

#### 3.1.2. Migrant Generations and Countries of Origin

While the research question was set out to explore the experiences of HCPs in the interaction with both first- and second-generation migrants, the HCPs reported almost exclusively on their experiences with first-generation migrants. Apart from very few exceptions, the second-generation migrants were not mentioned as patients, but rather in the treatment situation where they supported their parents during the visits to and the interaction with the HCPs. Hence, gender aspects in the interaction with second-generation were also rarely addressed or mentioned. Therefore, the term *migrants* is used in the following section of this paper to address first-generation persons with a migrant background. The HCPs did not always specify the countries of origin of their patients. The countries that were mentioned most often were, first and foremost, Turkey, followed by countries from the Arab regions. Patients’ affiliations with the Islamic faith were also frequently mentioned. Only very few statements concerned gender aspects in the interaction with people from other religious or regional backgrounds.

### 3.2. Challenges

Most statements addressed challenges. Within this main category, gendered issues affecting access to the treatment or care situation were most prominent, followed by more general challenges regarding the understanding of health-related information. No statements related primarily to the influence of gender on the processing step of applying health information.

#### 3.2.1. Gender-Specific Challenges Regarding Access to the Treatment Setting

##### Husbands as Gatekeepers

Some HCPs reported situations in which their interaction with migrant women was controlled or even prohibited by the women’s husbands. In some of these cases these observations were made regarding migrants from Turkey or the Arab region, but often the origin of the persons involved was not specified. While the husband’s motivation to control access to his wife was not always clear to the HCP, in part of the cases his aim was to ensure his wife would not be treated by a male HCP (see the category the gender of HCP as a factor). In some situations, the husband’s intervention lead to the termination of the treatment.

*The husband was not physically present, but then he practically forbade me to talk to the wife, because he must know everything. So, confidentiality does not occur in their thinking. So that the midwife discusses something confidentially with the woman, he as the father of the child, he must know everything, so, no. That was not possible at all. Moderator: How did you solve the situation in the end? HCP: I was not allowed to come any more. He prohibited it.*

– Midwife/Pediatric nurse (female, 45–54 years)

##### The Gender of HCP as a Factor

The gender of HCP was mentioned as a factor limiting interactions with migrant patients, mainly due to the patient’s need for an HCP of the same sex. While the HCPs reported gender concordance in the treatment setting to be important for both migrant men and women, they elaborated on it mostly with regard to women, who were seen as reluctant to be treated or cared for by male HCP. This was mostly related to persons of Arab or Turkish origin. Female HCPs reported that their expertise as an HCP was in some cases questioned by male migrants, especially from Russia, who favored male HCPs. In several descriptions, the gender of HCPs also influenced the role of migrant women’s husbands as gatekeepers who sometimes blocked contact of male HCP with their wives. In those cases, the gender constellation male HCPs–female patient–male husband led to complications regarding access to health care.

*And she definitely needed help, so she wouldn’t have come to the bathroom on her own, she wouldn’t have come to the toilet and so that dragged on for days until you were allowed to do more than just catlick and there really was the husband who was always in the room and always intervened somehow when a male nurse or doctor was there. So that was already difficult.*

– Nurse (female, 25–34)

##### Shame in the Health Care Situation

From the perspective of HCPs, shame of nudity was seen as a barrier for examination. This was not specified for migrants from certain regions or migration generations but for women the HCP assumed to be of Islamic faith, who were described as reluctant to undress. This regarded mainly two sorts of situations: Examination and washing of patients. Shame mainly harmed the access component of health literacy because it hindered examination, thus preventing the HCP from providing qualified health-related information for the patient. In addition, it was described as affecting understanding, for example if a low level of a person’s language proficiency made it more difficult for her to understand the HCP and to explain herself to the HCP. Dealing with these situations was considered time-consuming.

*So there are cultural things, [for example] undressing of strict, older Muslim ladies, you can forget it. Also, one must honestly say, temporarily, in the beginning I did it, but it costs half an hour of persuasion and then they stopped after the first layer. So, unfortunately that’s how it is, so I have to deal with it.*

– Physician (female, ≥ 55 years)

#### 3.2.2. Gender-Specific Challenges Regarding the Understanding of Health Information

##### Gender-Specific Aspects of Language Barriers

Many statements dealt with how the HCP perceived migrant women’s roles within the family. Especially for women of Turkish or Arab origin, aspects of these roles were often perceived as a limiting factor for the women’s ability to access and, more than anything, understand health information. Most of these statements regarded first-generation migrant women of Turkish origin who migrated to Germany in the 1960s and 1970s. They were mentioned as suffering from loneliness after a long time of raising children and doing housework, sometimes showing a fatalistic or indifferent attitude to their own health. The HCP talked about the situation of these women less by describing case histories than in a general way and also related to their own emotional processes of frustration or empathy. Level of education and German proficiency within this group of female migrants was perceived as low, partly due to the fact that they originated from rural areas with little educational infrastructure. While their male counterparts were also affected by this, they were described as being more in contact with persons of the German majority population due to their working experiences, which enabled them to acquire a certain level of language proficiency.

*So these Turkish women in particular, now, 50, 60 years old, children brought up, hardly any knowledge of German actually, also relationships lived, but basically also a lot of oppression so and now alone actually […].*

– Physician (male, 35–44 years)

#### 3.2.3. General Challenges Regarding the Processing Step of Understanding Health Information

##### Language Barriers

While there was a gender aspect regarding language barriers in the case of the elderly Turkish women, HCP described language barriers to generally impede the exploration of medical problems of migrant patients regardless of their religion and region of origin, also hindering the transfer of important information to them. This fact was seen as detrimental for a proper treatment.

*This considerable language barrier makes it of course difficult then to do the anamnesis and properly inform the patients legally, to carry out an intervention at all if it is not an acute emergency, and then of course the proper treatment is delayed.*

– Physician (female, 25–34 years)

##### Systemic Lack of Time

HCP described a systemic lack of time due to factors as lump-sum fees, personnel shortages or the undersupply of areas with low socio-economic status as a major general problem, hindering them from taking the individual’s needs into account. This was described as especially problematic in the treatment of migrants regardless of their respective origin or religion. Time pressure interacted with language barriers. For example, the amount of information transmitted within a given time frame could be smaller when language barriers slowed down communication. The HCP stated understanding the patient’s exact needs and overcoming gender-specific barriers as time-consuming and hardly feasible under the given circumstances. A physician described his way of treating young migrant men who suffered from sexual potency problems. He reported prescribing drug therapy in such cases although he did not consider it the optimal treatment. Finding out about the cause of the problem would take more time than the HCP said he was able to spend:

*With young men it is rarely an organic problem, it is more of a psychological problem. But you shouldn’t forget that a doctor’s office also means an average of five minutes of medicine. So now I can’t sit down with a young man who presents this problem and say, now I take half an hour for him and listen to exactly where the problem is. Then the waiting room would overflow.*

– Physician (male, ≥55 years)

#### 3.2.4. Gender-Specific Challenges Regarding the Appraisal of Health Information

##### Skepticism towards Psychotherapy

In general, HCP reported skepticism towards psychological issues and psychotherapy as common among migrants and often mentioned, especially regarding persons of Turkish or Arab descent. On the one hand, these patients were described as favoring somatic explanations to health problems that were or could be of a psychological nature. On the other hand, some HCPs also mentioned the importance of spiritual support. Skepticism towards psychological issues was seen as more prevalent in male than in female migrants and sometimes attributed to a traditional approach to masculinity obliging men to be physically and mentally strong and healthy breadwinners.

*I often hear that from patients, the Turks, who come to us to visit the psychiatrist, that is the very last alternative, if nothing at all works anymore. Those who try everything else, go to the imam; they don’t believe in psychiatric diseases.*

– Nurse (female, 35–44 years)

##### The Importance of Motherhood

The HCP observed that motherhood was a topic of major importance for migrant women of Turkish and Arab origin, more so than for women of the majority of the population. This was mainly connected to the appraisal of health information because information related to motherhood and pregnancy was considered much more relevant and valued more highly by migrant women than by women of German origin. The HCP also mentioned the necessity to gain a thorough understanding of the meaning of motherhood for migrant women in order to address the needs connected to its high priority. One physician also related this to the problem of systemic lack of time which kept her from learning more about this issue:

*For example, we are dealing with women who have pain during sexual intercourse, and a Turkish woman who has pain during sexual intercourse or an Arab woman who has the expectation to become pregnant immediately after marriage, otherwise something is wrong, is something completely different than with a woman who perhaps has a vaginal infection. So I wish for that, but it belongs to the many things that I would like to learn, [but] for which I also probably don’t have enough time.*

– Physician (female, 45–54 years)

### 3.3. Needs

The HCPs reported their needs for solving gender-related issues within the health care setting almost exclusively on a general level addressing the understanding of health information. These needs were not limited to interactions with migrants of specific regions of origin or religions.

#### 3.3.1. General Needs Regarding the Processing Step of Understanding Health Information

##### Cultural and Language Mediation/Interpretation

The need for interpretation services was stated repeatedly by the HCPs, although some expressed reservations concerning the greater need for time that could be caused by the interpretation process. Sometimes the participants combined this with the wish for those services to be covered by statutory health insurance. In several statements, the HCP wished for interpreters to act as cultural mediators as well. It was also stated that interpreters should have at least a basic level of medical knowledge.

*Language is totally important, and I have just thought about it, we are always at the point to demand that there should be language mediators in this area [...] Actually, it would be right for the health insurance companies. The health insurance funds would reduce health costs if, I believe, they were to finance language mediators so that doctors could use them locally, etc.*

– Other HCP (female, 35–44 years)

##### Need for More Time

While systemic lack of time was stated as a general challenge, the need for more time when dealing with persons with a migrant background played an important role as well. Time was said to be needed for overcoming language barriers but also for building a trusting relationship between HCP and patient. In this regard, the patient’s appraisal of health information as coming from a trusted source was also connected to the need for more time.

*That, I think, is also such a general topic, time, so that is something I perceive quite fundamentally, […] I really need much more time to explain things […].*

– Other HCP (female, 35–44 years)

### 3.4. Solutions

The applied solutions described were related to general issues concerning the interaction with migrants as well as to challenges that had a gender-specific aspect to them. Similar to the challenges stated by the HCP, the statements related to the processing steps access, understand and appraise.

#### 3.4.1. General Solutions Regarding the Processing Step of Understanding Health Information

##### Cultural and Language Mediators/Interpreters

Many HCP who had already worked with interpreters described this as helpful for the mutual understanding of HCPs and patients. On the other hand, some HCPs found the presence of a third person to complicate the relationship with the patient and slow down communication. All in all, interpreters were regarded as helpful for improving communication regardless of the genders of HCPs and patients, with no differences being stated for migrants of certain origins or religions. In one case regarding a refugee woman, consulting a remote video interpretation service helped to solve a misunderstanding concerning gender roles. Here, the husband’s role as a gatekeeper preventing his wife from leaving the house had merely been assumed by the HCP.

*We currently have a mentally ill pregnant woman, and we thought all the time, she is not allowed to go out and she is so mentally impaired that she does not go out alone, but then* [it occurred] *in a conversation that she does not know it from home, that they live in such a group of houses, inside is a yard, where the women meet, where the women move, but outside this yard, they don’t go anywhere and so she can’t find her doctor and doesn’t come to any psychologist and we had thought the whole time, the man doesn’t want that and then we had a video interpreter with us and then it came out that the man is actually completely open and just his wife isn’t used to going any ways alone.*

– Physician (female, ≥55 years)

#### 3.4.2. Gender-Specific Solutions Regarding Access to Health Information

##### Covering Parts of the Body to Mitigate Shame

In some cases, HCPs reported that Muslim women covered parts of their bodies during examination or care, sometimes using blankets provided by the HCP for this purpose, sometimes wearing full-body suits when they were washed. Those solutions were found to be feasible, despite being cumbersome and time-consuming workarounds.

*I notice for example that Germans sometimes [...] they come in and take off everything from bottom to top, [...] really naked. The Syrian or Iraqi or Muslim woman usually doesn’t do that. [...] So first she is ashamed and when I say, for example, on the chair, I will examine the breast now, then she says “No, first I dress from below”, so that she has a feeling, half of it is already covered and then she undresses the upper body.*

– Physician (female, ≥55 years)

#### 3.4.3. Gender-Specific Solutions Regarding the Appraisal of Health Information

##### Women as Pioneers for the Acceptance of Psychotherapy

Although the HCP found men of Turkish or Arab descent to be particularly skeptical about psychotherapy, some reported to observe a paradigm shift in that regard, with men belonging to this group slowly developing acceptance for this kind of treatment. Within this process of reappraisal, women were sometimes described to act as pioneers.

*The first ones with a migrant background were Turkish women brought by their daughters. […] And that’s really a development, until it came gradually that oriental men also came with the feeling that they had a psychological problem and you had to talk about it.*

– Physician (female, ≥ 55 years)

## 4. Discussion

This qualitative study explored the perceptions of health care professionals of gender aspects of their interactions with migrants mainly from Turkey and Arab countries. The interrelated challenges, needs and applied solutions were analyzed from a health literacy perspective. By relating the statements of HCP to the processing steps of health literacy, gender-specific challenges could be identified primarily regarding the access to health care and the appraisal of health-related information. Described needs and applied solutions mainly concerned mutual understanding between HCP and migrants.

Most of the statements concerned challenges the HCP experienced when dealing with persons with a migrant background. Three main gender-specific challenges related to the access to health information emerged from these statements: Husbands as gatekeepers regulating access of their wives to health care, the gender of HCP as a factor that could keep migrant women from receiving treatment or care from male HCPs, and shame in the health care situation hindering proper examination especially of Muslim women. The HCP rarely tried to provide explanations for such situations. Even though such situations seemed to occur mainly in the interaction with patients who were identified as Muslims by the HCP, they did not speculate on the exact role of religion in these cases. This may illustrate a lack of knowledge about Islam on part of some HCPs, but it was also the case for HCPs of Arab or Turkish origin who were more familiar with this religion than their colleagues. From the HCPs’ statements, it seemed that religion was usually not addressed directly in the treatment situation. Thus, many relevant aspects remained unclear, such as whether the patient was indeed a Muslim, what Islamic subgroup he or she belonged to or how important religion was to the patient. With gender equity being comparably low in most predominantly Islamic countries [48], relating gender aspects in the interaction with migrants to their religion may be tempting but probably a premature conclusion. For example, regulating the gender relations is not exclusive to Islam but can be found in many religions including Christianity, usually putting men in the more powerful position [49]. In a highly secular country as Germany, some observations of gender aspects might be misunderstood as specific to persons of Islamic faith while they may rather be connected to religiosity in general. The understanding of health as an individual matter, as it prevails in Germany, is not shared in many countries, especially not in Islamic regions, where the health of a person is often perceived as a family affair [50]. Thus, Muslim husbands may feel responsible for the health of their wives in a more pronounced way than non-Muslims [51]. Also, mistrust in authorities was sometimes mentioned by the HCP regarding migrants from countries with authoritarian political systems. This might also play a role in the protective behavior observed in some migrant husbands. There is some evidence for the importance of gender concordance with the HCP for women of Islamic faith in general [52] and for Turkish women in Germany [53] as well as for a restraint in Muslim women concerning nudity [54,55]. Again, the HCPs did not elaborate on how religion might influence the women’s perceived preferences. These categories describe that cultural differences in the personal determinant of gender were observed to primarily influence access to health care and health-related information. They also show that gender may be a personal determinant, but its impact on health literacy within the health care situation depends on the genders of all persons involved and on their respective interpretations and expectations regarding gender roles. These findings underline the social and relational character of health literacy.

The general subcategory Systemic lack of time describes a phenomenon well known in health care [56,57]. On average, primary care consultations in Germany last only 7.6 min; in a current systematic review, this was found to be one of the shortest durations among Western industrial nations [58]. Systemic lack of time can be interpreted as an omnipresent stressor concerning all actors within health care, with particular effect on the interactions with migrant patients. Research in social psychology has shown that people resort to stereotypes under time pressure [59]; this might have influenced the HCPs’ perceptions and descriptions of the interactions with migrant patients as well. With systemic lack of time as a backdrop, the second general challenges subcategory regarding the understanding of health-related information directly relates to a migration-specific issue: Language barriers. If communication was impaired due to language barriers, examinations and treatments were perceived to be compromised. This is in line with research demonstrating language barriers to be a serious disadvantage for migrants trying to obtain health care [60,61,62,63]. Time pressure seems to have an even stronger impact when it comes to dealing with patients who need more time due to the necessity of overcoming language barriers. This impact is further reinforced by gender-specific aspects of language barriers. The comparably low level of German proficiency within the group of elderly Turkish women has already been documented by researchers in Germany [64]. Additionally, the HCPs reported a high prevalence of depressive symptoms combined with a rather dismissive attitude towards psychotherapy within this group. This corresponds to current research which identified first generation migrant women from Turkey as especially vulnerable for depressive disorders [65] and skepticism towards psychotherapy to be more common in first- and second-generation migrants from Turkey than in the general population [66]. This connects to further gender-specific challenges which could be identified regarding the processing step of appraising health information. Although skepticism towards psychotherapy is generally known to be more common in men than in women [67,68], the HCPs mentioned it especially regarding men from Turkey and Arabia. Some saw a part of these men to favor a more traditional interpretation of masculinity, which has been found to be common for example in Turkey [69], and which is connected to a tendency to reject psychotherapy [70]. On the other hand, the availability of insurance-covered psychotherapy is special to Germany [71] and not common in Turkey [72]; unfamiliarity with the method may contribute to the skepticism against it. The importance of motherhood the HCPs observed in migrant women was regarded as a minor challenge, illustrating how persons from different cultures may evaluate the same piece of health information differently based on the relevance it has to their lives. In Germany, voluntary childlessness is much more common than in other countries [73], so that the significance of motherhood for migrant women could be an indication of the special situation in Germany rather than a particular feature of migrant women.

Gender aspects seem to act as a reinforcing factor for the general time problem within health care in Germany. In the case of migrants, overcoming language barriers takes time. If these barriers are higher, for example due to gender-specific reasons as in the case of the elderly Turkish women, communication takes even more time. In case it is necessary for these patients to undress in the health care setting, shame may additionally slow down the process. If the HCP is a male person, shame may play an even more important role and can stall the process even further. These phenomena were mostly seen in specific subpopulations, and we may not be able to understand them without considering cultural and religious aspects that should be analyzed in further research.

The HCP did not report on the needs for specifically solving gender-specific challenges. Instead, they almost unanimously addressed the needs for more time and for cultural and language mediators/interpreters. This suggests that the HCP see the processing step of understanding health-related information as the key health literacy element in the context of migration. Importantly, in most cases understanding was described as a mutual process—understanding the patient as well as making oneself understood by the patient. Meeting the need for more time may be to the benefit of migrant patients and to that of the whole population; doctors giving more time to the individuals instead of doing “five minutes of medicine”, as one physician put it in an FGD, would serve the HCP as well as the patients [74,75]. The shortage of HCPs in Germany is a widely discussed situation [76,77] which still does not seem to improve substantially [78]. Our research is in line with these observations. In the context of ongoing migration, the effects of this problem are particularly evident.

The HCPs also reported on applied solutions to solve the challenges they had elaborated on. Regarding *access* to health care, the gender-specific solution of covering parts of the body to mitigate shame of Muslim women was seen as a feasible, albeit cumbersome solution. As a general solution for addressing the processing step of understanding, some had already worked with cultural and language mediators/ interpreters, most of them reporting positive results, which is in line with studies focusing on the effectiveness of interpreter services [79,80,81]. Although being a general solution, this could also help with gender-specific aspects of language barriers. Regarding the processing step of appraisal, the gender-specific solution women as pioneers for the acceptance of psychotherapy seems especially remarkable in several ways, as it is (a) a solution coming from the migrants themselves and (b) an example for the (self-) empowerment of women being advantageous to men as well.

The three concepts gender, migrant background and health literacy can be understood very differently [82,83,84]. Within the FGD, participants addressed gender using the man-woman dichotomy with a strong emphasis on gender roles. The usage of the term migrant background was slightly different from the definition introduced by the moderators, because the participants usually referred to first generation migrants (as opposed to first- and second-generation migrants). In some respects it can be justified to examine the diversity of migrants in Germany as a group instead of focusing on certain subgroups. This is the case when it comes to phenomena associated with transnational migration in general, such as the need to find orientation in an unfamiliar health care system or to communicate in a new language. Looking at migrants in general can also reveal aspects that are special to the host countries instead of ascribing differences between migrants and non-migrants to culture, religion or other attributes of a certain migrant group. In contrast, the term migrant background, which is very common in German administration and research, covers people with and without a direct migration experience and is therefore known to be a controversial concept [85]. As the HCPs in the FGD used it almost exclusively for first generation migrants, the term seems dispensable at least for the purpose of this study. Furthermore, the HCPs often focused on patients of Turkish or Arabic descent. Most migrants living in Germany are of Turkish origin, and refugees from Syria and Iraq came to Germany in large numbers in recent years [86]. Although there are more people of Polish origin in Cologne than there are people of Iraqi, Syrian, Algerian, Moroccan, Libyan and Lebanese origin combined [87], not a single statement referred to persons of Polish origin. Migrants from Russia were only mentioned in connection with female health care representatives feeling rejected by male patients. It may be the case that the HCPs had only few encounters with patients of Polish origin, but this may also pose the question who is regarded as having a migrant background at all [88]. Additionally, only one of the HCPs was of Eastern European origin, but eight HCPs had roots in Turkey and Arab countries and reported to dealing with many patients from these regions, which may have contributed to focusing on these migrant groups during the FGD. Unsurprisingly, the term health literacy was rarely mentioned literally. Health literacy is a very broad concept; in real-life situations its determinants and processing steps may be observed rather than health literacy as a whole.

Regarding the health literacy model by Sørensen et al. [24] the allocation of statements to the processing steps was never a clear-cut decision, as these steps may overlap and interact. Our study partly questions the sequential nature of accessing, understanding, appraising and applying health-related information as proposed by Sørensen et al. [24]. From the perspective of the HCP, the negative appraisal of psychotherapy especially by migrant men prevented them from accessing information about this way of treatment. This is consistent with psychological research, which has shown the interrelatedness of perception and appraisal on multiple occasions [89,90]. Mutual understanding, improved by involving an interpreter, can eliminate false assumptions as in the case of the erroneously assumed role of a husband as a gatekeeper (see 3.2.1). This case also shows that understanding can also enable access. Furthermore, the health literacy of the HCP interacted with that of their migrant patients. A good example for this interaction is the processing step of understanding health information: By far the most statements in this regard were directed at reciprocal understanding. The ability to understand the patients and the ability of the patients to understand the HCP are mutually dependent. This emphasizes the social-relational nature of health literacy as well as its process character that already has been called “doing health literacy” [91].

By mapping real-life situations from the perspectives of HCPs to the integrated model of health literacy by Sørensen et al. [24], our research contributes to a deeper understanding of cross-cultural health care situations. Our findings suggest that challenges regarding the appraisal of health-related information may be connected to needs and solutions directed at a different processing step, namely understanding. To our knowledge, this is the first study exploring gender-specific aspects of health literacy of migrants from the perspective of HCP. A specific strength of our research lies in the application of the health literacy model by Sørensen et al. [24] to qualitative data with a concrete assignment of statements to the respective steps of health information processing. As far as we know, this has not yet been explored and can help to understand the complex relationships between systemic factors and gender aspects in the context of migration. Furthermore, our findings contribute to the further development of the concept of health literacy by (a) emphasizing the social-relational character of health literacy and (b) describing its processing steps as iterative rather than sequential elements. Another advantage of this research lies in the composition of the FGD. The participants were HCP from different professions, covering a wide range of ages and including first- and second-generation migrants as well as non-migrants.

There are several limitations to this study. First, it might be the case that the research question provoked generalizations about the diverse group of migrants. Asking the participants to refer to specific situations was aimed at preventing this. This may not have worked in every case, as relating to specific situations can mislead in regarding them as typical or representative for the migrant group mentioned. Second, the observations reported in this study may evoke stereotypes about persons of Islamic faith, a matter we intensely discussed within the research team. Although the participants of the FGD spoke with great empathy for migrants and more than 50% of them were first or second generation migrants themselves, it cannot be ruled out that stereotypes about persons of Islamic faith, for example about male Muslims [92,93] shaped some of their statements as well. With anti-immigration and anti-Islamic movements rising all over Europe [94,95], this is a delicate ethical matter, especially for researchers positioning themselves as favoring openness and equity. It is crucial not to see possible biases in their perceptions as personal deficits of the HCP. Stereotypes belong to the cognitive toolbox of all persons [96]. Overcoming them is especially difficult when acting under time pressure as it is the case in health care. The HCPs reported situations in which migrant women experienced serious health care disadvantages the HCP related to gender roles. We think these findings are important and should be reported. There may be situations that demand that HCPs act against perceived gender taboos in order to ensure adequate health care, especially for women, and there may be cases when doing so would do more harm than good. These difficult decisions have to be made by the HCP in every single case, and they clearly stated they need support in doing so. They strongly called for measures to improve mutual understanding with migrants. This indicates that they saw incomprehension and misunderstandings on both sides as the main causes of the challenges they perceived in interacting with migrants. Third, qualitative research is not aimed at representativeness [97]. This is also true for this study, as neither the participants are a representative selection of HCPs, nor the situations they described can be considered representative for the interaction with migrants. In most cases, gender-specific observations made by the HCP were limited to migrants from Turkey and Arab countries. Finally, it has to be mentioned that the FGD were held in the German language and translated into English. That may lead to a loss of information and/or bias in the meaning of the translated statements as they are presented in this manuscript.

Exploring the challenges, needs and applied solutions with regard to achieving optimal health care within different subpopulations of migrant men and women by letting them state their own perspective was outside the scope of this project. From our view, this would be the logical next step for further research in order to gain a more complete picture about gender-related aspects of health literacy in interactions with migrant patients.

## 5. Conclusions

Our research provides insights into the special role of gender in health literacy as perceived by HCPs when interacting with migrant patients mainly from Turkey and Arab countries. These results only represent the possibly biased or assumption-based perspectives of health care professionals on their migrant patients. From the HCPs’ point of view, gender-specific challenges can result in consequences for the way in which health-related information is accessed, understood, and appraised in cross-cultural health care situations. It also shows that meeting these challenges by reducing time pressure and providing resources for improving communication may help HCPs to better understand the individual needs of their patients and prevent them from using heuristics that can be associated with stereotyping. This may be to the benefit of all actors within the health care sector—HCPs as well as persons of all genders and countries of origin. The results of our study can sensitize HCPs and policy makers to gender-specific challenges in the cross-cultural health care settings and show possible starting points for their solutions, especially at the level of mutual understanding of HCPs and migrants. Further research should focus on the perspective of the migrants themselves, considering the specific situations of different groups from different countries of origin.

## Figures and Tables

**Table 1:** Main characteristics of the health care professionals (HCPs) participating in the focus group discussions (FGD) (*n* = 31).

Gender		Men	Women
Age (years)	25–34	1	4
	35–44	4	7
	45–55	5	3
	≥55	5	2
Migrant background	migrant background ^1^	8	8
	no migrant background	7	8
Occupation	physicians	8	5
	psychologists	1	1
	midwife/pediatric nursing	0	2
	nursing care	3	2
	Other HCP	3	6
Total		15	16

Note. ^1^ Regions of origin of HCPs with a migrant background were Turkey (*n* = 6), Arab region (*n* = 3), Central Europe (*n* = 2), South Europe (*n* = 2), Eastern Europe (*n* = 1), Asia (*n* = 1), Sub Saharan Africa (*n* = 1).

**Table 2:** Main categories.

Main Categories ^1^	Processing Steps ^2^	Gender Subcategories ^3^	General Subcategories ^3^
Challenges	Access	Husbands as gatekeepers	
		The gender of HCP as a factor	
		Shame in the health care situation	
	Understand	Gender-specific aspects of language barriers	Language barriers
			Systemic lack of time
	Appraise	Skepticism towards psychotherapy	
		The importance of motherhood	
Needs	Understand		Cultural and language mediation/interpretation
			Need for more time
Applied Solutions	Access	Covering parts of the body to mitigate shame	
	Understand		Cultural and language mediators/interpreters
	Appraise	Women as pioneers for the acceptance of psychotherapy	

Note. ^1^ Categories deductively derived from the objective of the study. ^2^ Subcategories deductively derived from the guiding model (Sorensen et al., 2012). ^3^ Subcategories inductively generated from the statements of the HCP.
